# A Compact Multi-Distance DCS and Time Domain NIRS Hybrid System for Hemodynamic and Metabolic Measurements

**DOI:** 10.3390/s21030870

**Published:** 2021-01-28

**Authors:** Caterina Amendola, Michele Lacerenza, Mauro Buttafava, Alberto Tosi, Lorenzo Spinelli, Davide Contini, Alessandro Torricelli

**Affiliations:** 1 Dipartimento di Fisica, Politecnico di Milano, piazza Leonardo da Vinci 32, 20133 Milan, Italy; michele.lacerenza@polimi.it (M.L.); davide.contini@polimi.it (D.C.); 2 Dipartimento di Elettronica, Informazione e Bioingegneria, Politecnico di Milano, via Ponzio 34/5, 20133 Milan, Italy; mauro.buttafava@polimi.it (M.B.); alberto.tosi@polimi.it (A.T.); 3 Consiglio Nazionale delle Ricerche, Istituto di Fotonica e Nanotecnologie, piazza Leonardo da Vinci 32, 20133 Milan, Italy; lorenzo.spinelli@ifn.cnr.it

**Keywords:** diffuse correlation spectroscopy, time domain near infrared spectroscopy, diffuse optics

## Abstract

In this work, we present a new multi-distance diffuse correlation spectroscopy (DCS) device integrated with a compact state-of-the-art time domain near infrared spectroscopy (TD-NIRS) device. The hybrid DCS and TD-NIRS system allows to retrieve information on blood flow, tissue oxygenation, and oxygen metabolic rate. The DCS device performances were estimated in terms of stability, repeatability, ability in retrieving variations of diffusion coefficient, influence of the tissue optical properties, effect of varying count rates and depth sensitivity. Crosstalk between DCS and TD-NIRS optical signals was also evaluated. Finally, in vivo experiments (venous and arterial cuff occlusions on the arm) were conducted to test the ability of the hybrid system in measuring blood flow variations.

## 1. Introduction

Diffuse optics (DO) is gaining more and more interest in biomedical research and clinical applications, for brain monitoring of adults and preterm neonates [1], for brain functional activations during cognitive and somatosensory tasks [2], for muscles oxidative metabolism assessment during exercise [3], and for spectroscopic tumor characterization [4]. Diffuse correlation spectroscopy (DCS) in particular allows to noninvasively measure microvasculature blood flow (BF) variations [5]. This technique exploits long coherence laser light that, in the near-infrared (NIR) region, penetrates a few cm inside human tissues and generates a speckle pattern (dark and bright intensity spots) at the collection plane, due to the constructive and destructive interference of photons scattered by different scattering centers. In the case of biological tissues in the NIR, red blood cells (RBCs) are responsible for the dynamic scattering process. Movement of RBCs causes the intensity of each speckle to fluctuate in time. By measuring the intensity fluctuations of a single spot and by computing its intensity autocorrelation function, DCS allows to quantify blood flow variations.

Many clinical studies have been performed, highlighting the strength of this technique. Some examples are the use of DCS techniques during clinical interventions, to assess cerebral autoregulation in ischemic stroke patients [6], or continuous (8-h) monitoring of cerebral blood flow (CBF) in comatose patients [7]. The DCS technique is commonly used in infants, obtaining a better reproducibility than in adults due to a higher SNR and reduced superficial layer thickness [6]. Recently, Giovannella et al. [8] studied the correlation between estimates of CBF by DCS and by positron emission tomography (PET) signals in animal model, and Diop et al. [9] measured the correlation between DCS signals and CBF changes measured by time resolved NIR instrument. These works pave the way for an absolute CBF monitoring by DCS.

To analyze DCS signal, tissue optical parameters need to be estimated [5], that is why recently different devices have been built combining DCS with frequency-domain NIRS (FD-NIRS) [10] or time-domain NIRS (TD-NIRS) [11,12,13]. FD-NIRS and TD-NIRS allow to estimate absolute values of absorption (µ_a_) and reduced scattering (µ’_s_) coefficients [2], and from them to retrieve absolute concentration of oxygenated hemoglobin (HbO_2_), deoxygenated hemoglobin (HHb), total hemoglobin (tHb = HbO_2_ + HHb), and also tissue oxygen saturation (S_t_O_2_ = HbO_2_/tHb). FD-NIRS exploits variations in amplitude and phase of modulated light injected in the tissue; TD-NIRS measures intensity and shape variations of laser pulses backscattered by the tissue. The use of hybrid instruments that combine DCS techniques with NIRS devices provides information that the two techniques separately are not able to retrieve, like the cerebral metabolic rate of oxygen consumption (CMRO_2_) [5,12].

In this work, we present a compact hybrid DCS instrument that embeds a state-of-the-art compact TD-NIRS device [14]. The DCS device presents short and long inter-fiber distances, in order to discriminate signal contribution of shallow layer from that of deeper tissues. The DCS device performances were assessed in terms of stability and repeatability, ability in retrieving variations of diffusion coefficient, influence of the tissue optical properties, effect of varying count rates, depth sensitivity. Crosstalk between DCS and TD-NIRS signals was also evaluated. Finally, in vivo experiments (venous and arterial cuff occlusion on the arm) were conducted to test the ability of the hybrid system in measuring blood flow variations and discriminating the response from superficial layer from deep tissue.

## 2. Materials and Methods

In this section, we present the developed device (Section 2.1), and we briefly summarize the physical principles at the basis of DCS techniques (Section 2.2). Finally, the characterization measurements, performed to assess the instrument performances, and the in-vivo protocols executed on volunteers are described in Section 2.3 and Section 2.4, respectively.

### 2.1. Instrument Description

**Figure 1:**
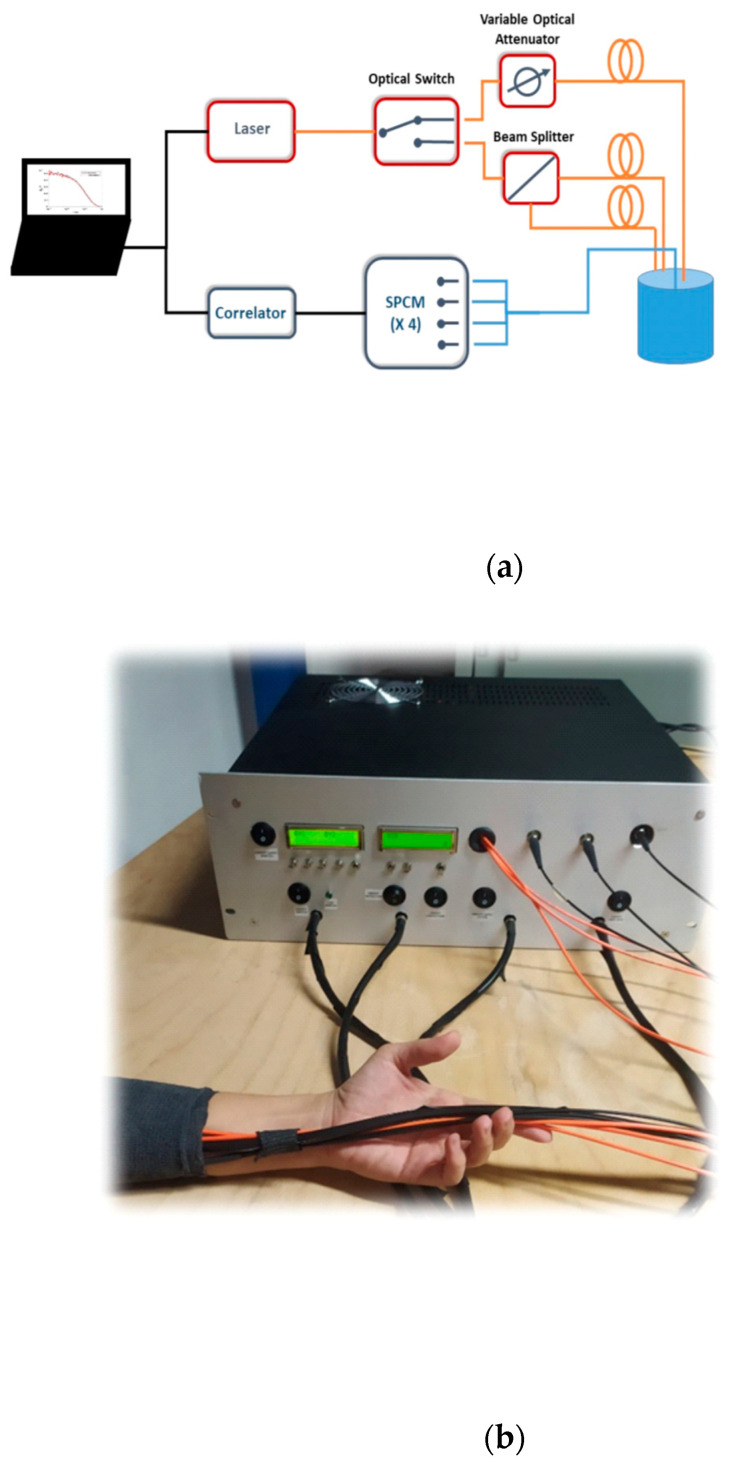
(**a**) A schematic of the Diffuse Correlation Spectroscopy (DCS) device we developed. (**b**) A photo of the instrument during an in vivo measurement.

**Figure 2:**
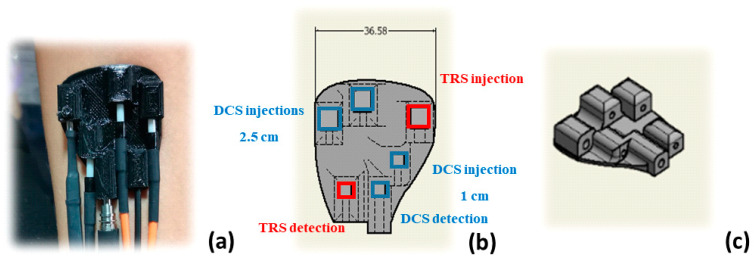
Panel (**a**) shows a photo (top view) of the probe developed for the in vivo measurements. Panels (**b**,**c**) show 3D draw, from bottom and lateral view, respectively. In panel (**b**), the dimensions of the probe are expressed in mm, red and blue boxes represent the Time Resolved Spectroscopy (TRS) and DCS injection and detection positions, respectively.

A schematic of the main optical and electronic components of the DCS system is reported in Figure 1. The injection part is composed of a highly coherent (>8 m coherence length) continuous-wave diode laser, operating at 784 nm (iBeam Smart, TOPTICA Photonics AG, Munich, Germany), with a nominal maximum power of 120 mW. Light emitted by the laser is coupled to a step-index glass optical fiber (100/125 µm core/cladding, OZ optics Ltd., Ottawa, Canada) and sent to an optical switch (mol 2 × (2 × 2), LEONI Fiber Optics GmbH, Jena, Germany), which alternatively directs the optical signal into two branches. In the first branch, light passes through an optical attenuator (DD-200-55-785-400/430, OZ Optics LTD., Ottawa, Canada), and it is then connected to a step-index glass fiber (400/430 µm core/cladding, OZ optics LTD., Ottawa, Canada) fixed on the optical probe, 1 cm distant from the detection position (see Figure 1). In the second branch, the light is divided into two more lines by an optical beam splitter (FOBS-12P-111-400/430, OZ optics LTD., Ottawa, Canada). The two output fibers (step-index, 400/430 µm core/cladding, OZ optics LTD., Ottawa, Canada) of the beam splitter end in the optical probe, 2.5 cm away from the detection position (see Figure 2). The light backscattered by the tissue is collected by a bundle of four single mode optical fibers (5 µm core, and NA: 0.13), and directed to four single-photon avalanche diodes (SPCM-AQRH-3XSPAD, Excelitas Technologies Corp., Miamisburg, OH, USA). The electrical signals generated by the SPAD become the input of a 4-channel digital correlator (ALV 70004USB/FAST, ALV GmbH, Hessen, Germany), which allows to retrieve the intensity autocorrelation functions of the four detector signals, in parallel. The autocorrelator shortest integration time is 1 s, with 3 ns delay-time, and 200 channels. The laser and the correlator are controlled by a PC. The optical switch and the attenuator are commanded by specific microcontrollers (dsPIC30F6014, Microchip Technology Inc., Chandler, AZ, USA). Synchronization between correlator and optical switch, and between DCS and TD-NIRS modules, is managed by a microcontroller through logic signals. The overall device is hosted in a 19” 4U module (see Figure 1b) with dimensions of 45 × 40 × 16 cm^3^, which also contains the compact TD-NIRS device developed at Politecnico di Milano which operates at 670 and 830 nm, with laser repetition rate of 50 MHz, detection active area of about 1.7 mm^2^, and temporal resolution of 10 ps (see [15] and [14] for further details). The DCS laser signal is blocked with a custom designed dual band pass filter in the detection chain of the TD-NIRS device.

The probe presented in Figure 2 has been 3D printed [16] with flexible material (PoliFlex^TM^, Polymaker, Suzhou, China), and hosts DCS and TD-NIRS optical fibers. It was specifically designed to perform in vivo measurements (see Section 2.4). Indeed, thanks to the 90° light deflection [3] (obtained with optical prisms, in both injection and detection paths) and its high flexibility, it allows to easily adapt and adhere to human skin. The optical probe is designed to firmly host the prisms and the fiber tips without the need of the glue. The probe material strongly adheres to the hosted objects preventing movements during measurements. According to EN-60825-1, the maximum power that can be safely injected in human tissues is 28 mW (for 0.39 NA fibers, 7 mm fiber to tissue distance, and 785 nm CW laser). Therefore, the optical signal of the longer inter-fiber distance was divided in two injection points (12 mm distant from each other), with maximum power of 28 mW each. The probe is composed of three injection points for the DCS module, two at 2.5 cm distance and one at 1 cm from the DCS detection, one injection point for the TD-NIRS module at 3 cm distance from the TD-NIRS detection. For measurements performed on liquid phantoms, a different probe was used, 3D printed with a black polylactide filament (PLA), which sends and collects the light directly to and from the phantoms without any prism.

### 2.2. DCS Data Analysis

In DCS technique, a highly coherent laser light is injected in the tissue and the intensity fluctuations of a single speckle, at the detection plane, are measured over time through the intensity autocorrelation function ($g_{2}$), related to the electric field autocorrelation function ($g_{1}$) through the Siegert relation [17]: $g_{2}(\vec{r},\tau )=1+\beta |g_{1}(\vec{r},\tau ){|}^{2}$ where $\vec{r}$ is the position vector, τ is the delay time, and β is a parameter depending on the collection optics of the instrument and on the number of detected speckles. The unnormalized electric field autocorrelation function ($G_{1}$) diffuses in the tissue similarly to the light fluence rate, so it obeys a correlation diffusion equation [18,19]. In case of homogeneous semi-infinite media, the solution of the diffusion correlation equation for $G_{1}$ is:

(1) $$G_{1}\left(\rho ,\tau \right)=\frac{vS_{0}}{4\pi D}\;\left(\frac{\exp\left(-K\left(\tau \right)r_{1}\right)}{r_{1}}-\frac{\exp\left(-K\left(\tau \right)r_{2}\right)}{r_{2}}\right),$$

where ρ is the inter-fiber distance, $S_{0}$ is the source intensity, $r_{1}={\left(\rho^{2}+{z_{0}}^{2}\right)}^{1/2}$, $r_{2}={\left(\rho^{2}+{\left(z_{0}+2z_{b}\right)}^{2}\right)}^{1/2}$, with $z_{0}=1/{µ’}_{s}$, $z_{b}=2\left(1+R_{eff}\right)/3{\;µ\;\prime }_{s}\left(1-R_{eff}\right)$ and $R_{eff}$ is the effective Fresnel reflectance. Finally, K(τ) =$\;\sqrt{3{µ}_{a}{µ’}_{s}+{µ’}_{s}^{2}k_{0}^{2}\alpha \langle \Delta r^{2}\left(\tau \right)\rangle }$, with $k_{0}^{2}$ the wavenumber, $\left\langle \Delta r^{2}\left(\tau \right)\right\rangle$ the displacement of scatterers at time τ, and α the fraction of moving scatterers over the total ones.

The motion of RBCs in microvasculature was approximated as Brownian motion, considering $\langle \Delta r^{2}\left(\tau \right)\rangle =6D_{b}\tau$ with $D_{b}$, the effective diffusion coefficient of the Brownian motion. In case of liquid phantoms α ≅ 1, whereas in case of human tissues, the exact value of α is unknown and the blood flow index (BFI) equal to α$D_{b}$ is used to estimate the blood flow.

All measurements were analyzed using Equation (1). The optical properties used in the analysis were retrieved with state-of-the-art TD-NIRS devices [14,20]. When all the four detection channels were used, the intensity autocorrelation functions of the four channels were first averaged and then the resulting curve was analyzed.

### 2.3. Characterization Measurements

The measurements we performed to characterize the DCS instrument are presented in this section.

#### 2.3.1. DCS System Behavior Over Time

Two kind of measurements were performed to test the DCS system response over time:

- Stability: a long measurement, about 3 h, with integration time of 1 s, was performed on a liquid phantom to test the possible trend of variations in the retrieved $D_{b}$ and β parameters over time. The phantom was composed by distilled water, Intralipid and black India ink. The percentage of Intralipid and ink were selected to mimic cerebral optical parameters (nominal values $µ{’}_{s}$= 10 cm^−1^, ${µ}_{a}$= 0.1 cm^−1^). The coefficient of variation (CV) was computed as the ratio between the standard deviation and the average value over all measurement times, $CV=\sigma \left(x\right)/x$ for the two parameters $D_{b}$ and β.
- Reproducibility: 30 measurements of 1 s integration time were repeated on the same liquid phantom, under the same experimental conditions, for 10 days. The liquid phantom used is a water-based solution of polydisperse microparticles (HemoPhotonics S.L., Barcelona, Spain) [21], it is not a biodegradable phantom, and therefore it does not change its composition over time, allowing us to reproduce the same measurements condition over different days. The $D_{b}$ was estimated using the mean optical parameters retrieved over the 10 days. To estimate the ability of our DCS system in reproducing similar results under the same experimental conditions, the CV was computed for the β and $D_{b}$ parameters retrieved on different days.

For these two measurements, the inter-fiber distance was set to 1 cm and only one detection channel was used. The optical properties were measured with the state-of-the-art system described in [20].

##### 2.3.2. $D_{b}$ Variations with Optical Properties, Temperature, Viscosity, and Count Rate

We studied also how $D_{b}$ parameter was influenced by variations of phantom composition, in terms of its viscosity, optical properties, and temperature. Indeed, from [22], the electric field autocorrelation function depends on the optical parameters, and their variation could affect $D_{b}$ estimation. Moreover, the $D_{b}$ depends both on temperature and phantom viscosity, as reported in the Einstein relation [23]:

(2) $$D_{b}=\frac{k_{B}T}{6\pi r\eta }$$

where *T* is the phantom temperature, *η* is the dynamic viscosity of the solution, *r* is the radius of the scattering particle inside the solution, and $k_{B}$ is the Boltzmann constant. Therefore, by changing *η* and *T*, variations on $D_{b}$ should be estimated by Equation (2).

Moreover, effects of signal intensity were tested by changing count rates of acquired signals.

For all these measurements, we performed 30 repetitions of 1 s acquisition time. Experimental conditions are summarized below, grouped on the basis of the varying parameter:

- Phantom viscosity: five liquid phantoms, made up of distilled water, lipofundin, ink, and glycerol were made. The glycerol concentration varied from 0 to 40% [24] in step of 10. Increasing glycerol concentration, the viscosity of the phantom became higher, and from Einstein equation (Equation (2)), we expect a decreasing $D_{b}$. The inter-fiber distance was 1 cm, and only one detection channel was exploited.
- Phantom optical parameters: eight liquid phantoms were prepared with different concentrations of Intralipid and black India ink, to change $µ{’}_{s}$ and ${µ}_{a}$, respectively. Measurements were first analyzed considering the average values of optical properties over all the phantoms, and then reanalyzed using the proper optical properties measured for each phantom with TD-NIRS instrument. The two results were compared, highlighting the errors done in the estimation of the $D_{b}$ parameter when wrong optical coefficients were used.
- Phantom temperature: the liquid phantom, made up of distilled water, Intralipid, and black India ink (0% glycerol), was heated up from 20 to 40 °C (in step of 10 °C). $D_{b}$ was estimated using the optical properties measured at each temperature. From Einstein relation (Equation (2)), we expect it to increase with temperature.
- Count rates: the probe was positioned on a liquid phantom, and measurements were acquired changing the power emitted by the laser. The total count rate for each detection channel was increased, and the correspondent $D_{b}$ estimated.

##### 2.3.3. Bilayer Measurements

The sensitivity of the DCS system to deep tissue was tested acquiring measurements on a phantom made up of two different layers [25]. The superficial layer was constituted by a liquid phantom made up of distilled water, lipofundin and ink, 0% glycerol. The thickness of the superficial layer was increased from 2 to 16 mm, in steps of 2 mm. The measurements were repeated using two phantoms with different viscosity in the second layer (10 and 20% of glycerol), and at two inter-fiber distances (1 and 2 cm).

##### 2.3.4. TD-NIRS Signal Crosstalk

The disturbances of the TD-NIRS signal on DCS measurements were studied. We placed our probe on a liquid phantom and simultaneous DCS and TD-NIRS acquisitions were performed. The TD-NIRS lasers [14] were operating at their maximum emission power (about 3.5 mW for each wavelength). Measurements were repeated, changing the distance between detection of the DCS system and injection of TD-NIRS in the range 10–42 mm.

### 2.4. In Vivo Measurements

Finally, measurements on volunteers were performed to test our instrument performances in retrieving BFI variations. All subjects included in these measurements cooperated voluntarily and previously provided written informed consent to the procedures of the study, which was approved by the Ethics Committee of Politecnico di Milano. The measurements were performed simultaneously with the hybrid DCS and TD-NIRS system [14]. The probe used is the one described in Section 2.1, with double inter-fiber distance for DCS (1 and 2.5 cm), and single inter-fiber distance of 3 cm for TD-NIRS. Acquisition time was 1 s for both devices. DCS signal was switched every 1 s from one inter-fiber distance to the other. By means of Beer’s law, HbO_2_ and HHb concentrations were derived from the absorption coefficients estimated with TD-NIRS, assuming a water concentration of 70%.

#### 2.4.1. Venous Occlusion

The hemodynamic properties of two volunteers (male, adults, 52 and 42 years old) were measured on their arm during a venous occlusion. The subjects were asked to sit, with their left arm laying on the table at approximately the same height of the heart. Maximum and minimum blood pressures were measured for each volunteer. The measurements protocol consisted of 1 min of baseline, 30 s of induced venous occlusion (a blood pressure cuff, positioned on the left bicep, was inflated at 100 mmHg to generate pressure between subject’s systolic and diastolic pressure), and 2 min of recovery (the cuff was deflated). The protocol was repeated three times for each subject and the three repetitions were averaged.

#### 2.4.2. Arterial Oclusion

Arterial occlusion is a well consolidated in vivo protocol widely used to validate in-vivo measurement performed by DCS instrumentations. Changes in blood flow are measured during an arterial occlusion, which is generated with a blood pressure cuff set above the systolic pressure. During the measurements, a blood pressure cuff was placed around the left bicep of the subject. The protocol consisted of 2 min of baseline (the subjects was asked to seat with the harm laying on a table, at approximately the same height of the heart), 6 min of occlusion (the pressure of the cuff was manually increased to reach 250 mmHg), and 5 min of recovery (the cuff was deflated and post arterial occlusion measurement acquired). The protocol was repeated three times for each subject (male, adults, 35 and 52 years old) and the results averaged over the repetitions.

## 3. Results

### 3.1. Characterization Measurements

#### 3.1.1. Stability

**Figure 3:**
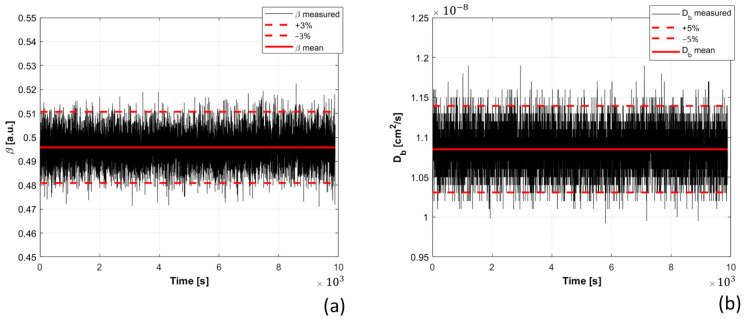
β (**a**) and $D_{b}$ (**b**) estimated during the stability measurement. In each panel, the red continuous line represents the average value estimated over all the measurements, the red dashed lines instead represent ±3% and ±5% ranges for β (**a**) and $D_{b}$ (**b**), respectively.

A 3 h measurement, under the same experimental conditions, was performed to assess the performance of the DCS instrument over time. Phantom optical parameters were measured [20], and used for the DCS analysis. The retrieved optical parameters were: µ’_s_ = 10.6 cm^−1^, µ_a_ = 0.11 cm^−1^. The results obtained for β and $D_{b}$ are presented in Figure 3. A small variation of $D_{b}$, lower than 3%, was observed during the first seconds (about 100 s). After this small warmup time, both β and $D_{b}$ parameters were constant during the measurement. The retrieved $D_{b}$ falls within ±5% (red dashed lines in Figure 3b), with a CV of 2.4%. Better results were obtained for β, which stays within ±3% (red dashed lines in Figure 3a), with a CV of 1.4%.

#### 3.1.2. Reproducibility

**Figure 4:**
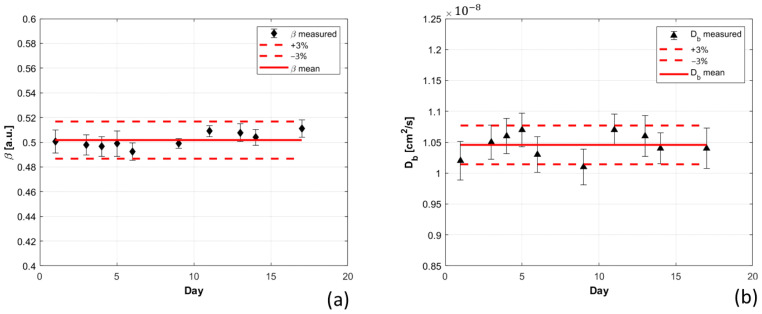
β (**a**) and $D_{b}$ (**b**) estimated over 10 different days. In each panel, the red continuous line represents the average value estimated over all the measurements, the red dashed lines instead represent ±3% ranges for both β (**a**) and $D_{b}$ (**b**).

A non-degradable liquid phantom was measured 10 times, over a total period of 17 days. Its optical parameters, estimated using the semi-infinite homogeneous model for photon migration, were averaged over the 10 measurements, obtaining: $µ{’}_{s}$ = 10.6 ± 0.3 cm^−1^, ${µ}_{a}$= 0.11 ± 0.001 cm^−1^. Using these values as optical parameters to fit the g_1_ autocorrelation function, β and $D_{b}$ were estimated, and the obtained results are shown in Figure 4. The dispersion of the retrieved values of $D_{b}$, and β over different days of measurements were always lower than 3.2% and 1.9%, respectively. Moreover, the computed CV was 2.0% for $D_{b}$, and 1.2% for β, highlighting the reproducibility of the measurements performed with our system.

#### 3.1.3. Phantom Viscosity

**Figure 5:**
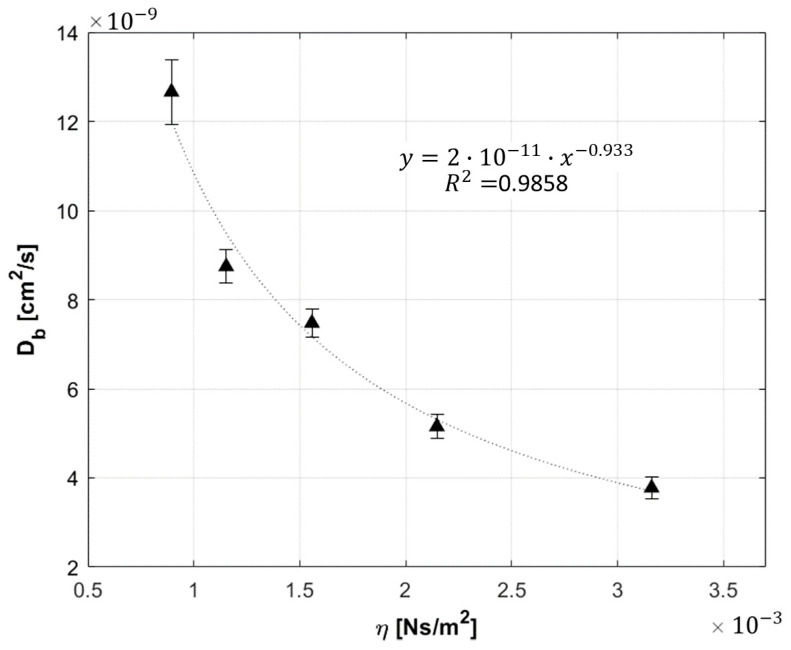
$D_{b}$ as a function of the phantom viscosity. The results were fitted with a power law (dashed black line), and R^2^ = 0.99 was obtained for a power of −0.93.

Phantoms with different viscosity were measured to test the ability of the DCS system in discriminating $D_{b}$ variations. The phantoms were prepared changing the glycerol concentration, and following the receipt reported in [24]. The $D_{b}$ value was estimated using the optical parameters measured for each phantom. The dynamic viscosity of the solution was retrieved as η = υ δ, where δ is the density of the phantom, and υ the kinematic viscosity of the solution at 25 °C, estimated from [26]. Due to the small amount of lipofundin and ink used (lower than 3% and 0.4% for lipofundin and ink, respectively), we neglected their contribution to υ, and we computed δ by approximating the phantom as composed just by water and glycerol. The results obtained are shown in Figure 5, where $D_{b}$ is almost inversely proportional to the estimated η.

#### 3.1.4. Phantom Optical Parameters

**Figure 6:**
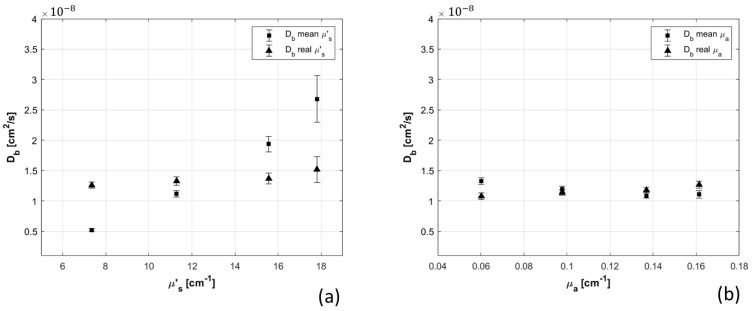
$D_{b}$ estimation through semi-infinite homogeneous model varying the scattering coefficient of the phantoms (panel (**a**)) and their absorption coefficient (panel (**b**)). $D_{b}$ has been estimated by using optical properties measured for each phantom (triangles in the two graphs (**a**,**b**)) and average optical properties over all the phantoms (squares in the two graphs (**a**,**b**)).

Optical and dynamic properties of phantoms with different concentration of Intralipid and ink were measured with TD-NIRS and DCS devices. This experiment was performed to highlight the importance of having correct estimation of absolute optical parameters, in retrieving accurate $D_{b}$ values. The optical parameters of the phantoms are reported in Table 1. The autocorrelation curves were analyzed in two different ways: using the mean optical parameters among all the phantoms (squares in Figure 6) and changing the optical parameters for each phantom (triangles in Figure 6). Differences between results of the two obtained datasets highlight the importance of the hybrid device for correct $D_{b}$ estimation. The error on the $D_{b}$ estimation performed using the average µ_s_’ is higher than the one obtained when average µ_a_ is considered. An error of 41% (45%) on µ_s_’ (µ_a_) generates an error of 76% (12%) on the $D_{b}$.

#### 3.1.5. Phantom Temperature

**Figure 7:**
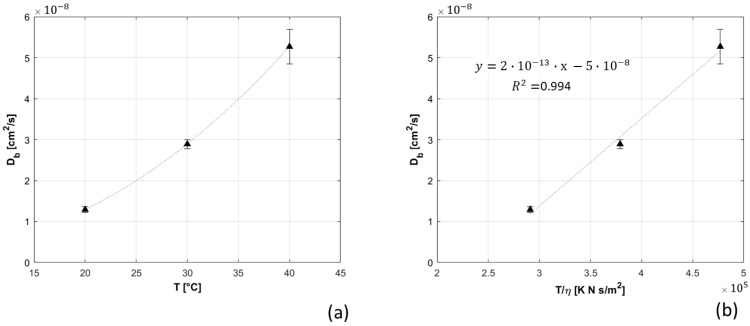
$D_{b}$ measured at different temperatures. In panel (**a**), $D_{b}$ is presented as a function of temperature; in panel (**b**), it is represented as function of ratio between temperature and dynamic viscosity.

The $D_{b}$ measured at 20, 30, and 40 °C is reported in Figure 7 as a function of T and of T/η, showing, as expected from Equation (2), a linear correlation with T/η with R^2^ equal to 0.994.

#### 3.1.6. Total Count Rates

**Figure 8:**
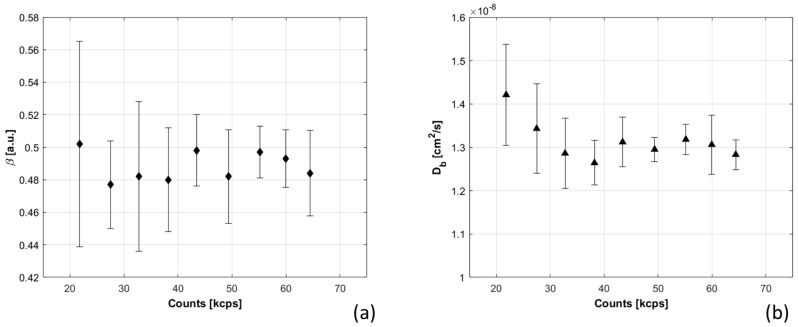
β (panel (**a**)) and $D_{b}$ (panel (**b**)) are shown as function of the average photons measured in each one of the four detection channels. The error bars are the standard deviations obtained for 10 repetitions.

$D_{b}$ and β parameters were measured when the count rate of the detected signal was varied. Figure 8 shows their trends: in the case of $D_{b}$, the increase of count rates corresponds to a reduction of the measured values together with a reduction of the error bars. A plateau is reached for the $D_{b}$ parameter when the count rates is higher than 40 kcps per channel. For what concerns β, no substantial variations have been noticed. We also analyzed the data using fixed β value, and we observed a large increase in $D_{b}$ errors, which suggests that better results are obtained by computing β for each autocorrelation curve.

#### 3.1.7. Bilayer Measurements

**Figure 9:**
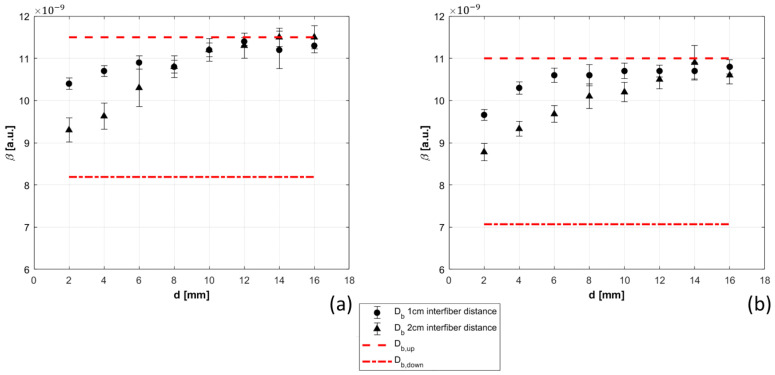
$D_{b}$ measured at 1 cm inter-fiber distance (dots in panels (**a**,**b**)), and 2 cm inter-fiber distance (triangles in panels (**a**,**b**)) in the bilayer phantom. $D_{b}$ was estimated increasing the thickness of the upper layer from 2 to 16 mm in steps of 2 mm (the *x* axis of the two panels (**a**,**b**). The measurements were repeated with two phantoms of different viscosity (10% glycerol in panel (**a**), and 20% glycerol in panel (**b**) in the bottom layer.

$D_{b}$ of the bilayer phantom described in Section 2.3.3 was measured at two inter-fiber distances (ρ): 1 cm (dots in Figure 9) and 2 cm (triangles in Figure 9), changing the thickness of the superficial layer from 2 to 16 mm in steps of 2 mm. The phantoms were created following the procedure described in [24], reproducing the same optical properties for all the phantoms. The measured optical properties are: for 0% glycerol µ_s_’ = 13.20 ± 0.11 cm^−1^, µ_a_ = 0.110 ± 0.001 cm^−1^, for 10% glycerol µ_s_’ = 13.3 ± 0.10 cm^−1^, µ_a_ = 0.112 ± 0.001 cm^−1^, for 20% glycerol µ_s_’ = 12.77 ± 0.1 cm^−1^, µ_a_ = 0.108 ± 0.001 cm^−1^. Results obtained analyzing the data with semi-infinite homogeneous model for electric field autocorrelation function are presented in Figure 9. Reference $D_{b}$ values of the two layers were obtained by measuring the corresponding homogeneous solutions and are reported as dashed red lines in Figure 9. Increasing the thickness of the first layer, the sensitivity of the second layer decreases and becomes almost zero for 12 mm thickness (≥ρ/2). As expected, the sensitivity to the bottom layer is lower when 1 cm inter-fiber distance is used.

#### 3.1.8. Influence of TD-NIRS Signal

**Figure 10:**
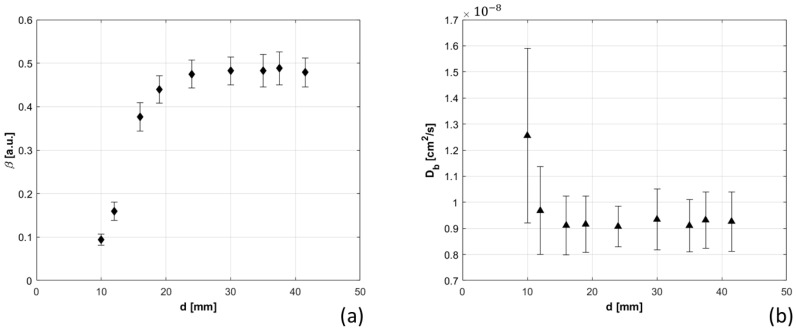
β (panel (**a**)) and $D_{b}$ (panel (**b**)) are shown as a function of the distance between the DCS detection point and Time Domain near infrared spectroscopy (TD-NIRS) injection position, at maximum power of TD-NIRS lasers (about 3.5 mW).

Interference of TD-NIRS signal in DCS measurements was assessed. The distance (d) between TD-NIRS injection fiber and DCS detection fiber was sequentially reduced, and the dynamic parameters were retrieved. Results are shown in Figure 10. It appears evident the reduction of β for small distances between TD-NIRS source and DCS detection, with consequent increase of the estimated $D_{b}$ and its error. $D_{b}$ reaches a plateau for 15 mm of distance, whereas longer distance needs to be reached in case of β, which becomes constant at about 20 mm.

#### 3.1.9. Venous Occlusion

**Figure 11:**
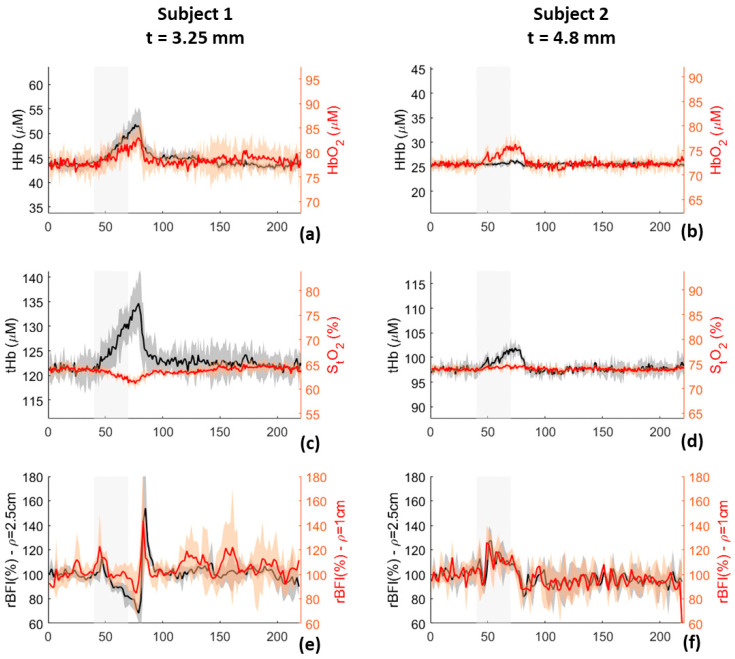
HHb, HbO_2_ (panels (**a**,**b**)), tHb, S_t_O_2_ (pannels (**c**,**d**)), and rBFI (panels (**e**,**f**)) measured during venous occlusion on the left forearm of two subjects. Black and red lines represent the average over the three repetitions done for each subject, shadows represent the range of variations over the three repetitions. t is the superficial skin thickness of the two subjects: 3.25 mm for subject 1 and 4.8 mm for subject 2.

The hemodynamic parameters of left arm of two volunteers were measured during a venous occlusion, with our hybrid device. In Figure 11, red and black solid lines are the average hemodynamic parameters measured in the three repetitions, and the shadows represent their range of variations (standard deviation over the repetitions). tHb and S_t_O_2_ are presented in panels c and d of Figure 11: tHb increases after the occlusion in both the subjects as expected [27], subject 1 showed larger variations of tHb with respect to subject 2, probably due to the lower thickness of the superficial layer. Concerning tissue saturation, a slight reduction was observed in subject 1, whereas no appreciable variations were measured in subject 2. In panels (e) and (f) of Figure 11, relative BFI (rBFI) is computed as the ratio between the measured BFI and the mean BFI during the baseline. A small increase of BFI can be observed for both subjects at the beginning of the occlusion, with a subsequent reduction during the occlusion. When the blood pressure cuff was deflated, a fast increase of BFI is observed in both subjects, which is more evident in subject 1.

#### 3.1.10. Arterial Occlusion

**Figure 12:**
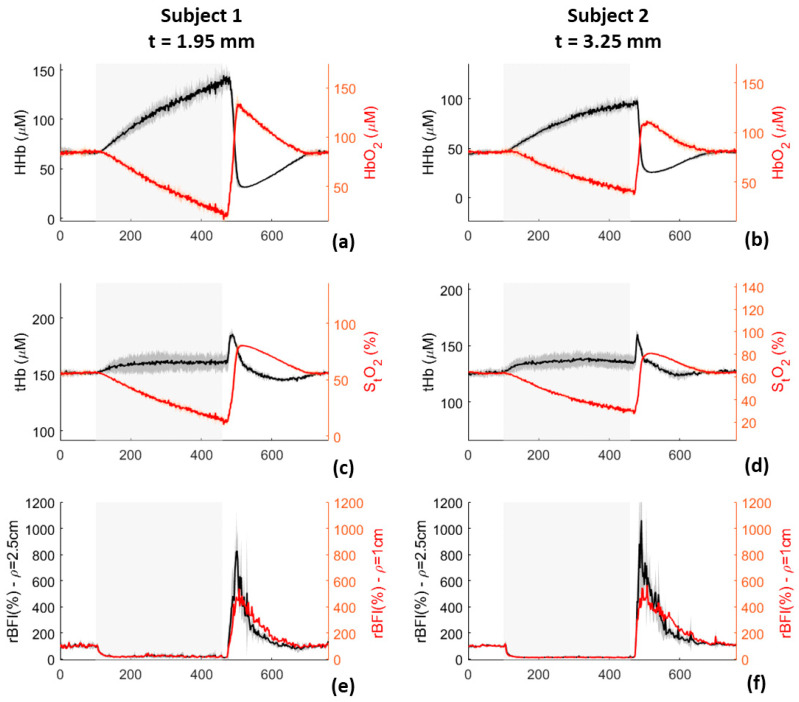
HHb, HbO_2_ (panels (**a**,**b**)), tHb, S_t_O_2_ (pannels (**c**,**d**)), and rBFI (panels (**e**,**f**)) measured during arterial occlusion on the left forearm of two subjects. Black and red lines represent the average over the three repetitions done for each subject, shadows represent the range of variations over the three repetitions. Superficial skin thickness (t) was reported for the two subjects: 1.95 mm for subject 1, and 3.25 mm for subject 2.

Arterial occlusion was measured with our compact hybrid TD-NIRS and DCS device. The results of TRS and DCS modules are shown in Figure 12. In addition, in this case, black and red lines represent the average values over the three repetitions, whereas the shadows are the range of variations over the three repetitions. Reduction of S_t_O_2_ and BFI was observed during the occlusion for all the subjects, in accordance with previous findings [12,28]. The S_t_O_2_ reduction is larger in the subject with a thinner layer of skin, as expected. In case of rBFI, for short inter-fiber distance, the increase estimated at the end of the occlusions present a faster response than the one observed for long inter-fiber distance. Moreover, variations of rBFI, when the occlusion was released, were higher for smaller inter-fiber distance.

## 4. Discussion

In this work, we presented our DCS system for BF monitoring. The DCS module was integrated with a compact state-of-the-art TD-NIRS system [14]. Hybrid instruments that combine multi-distance DCS with TD-NIRS modules have been already presented in previous works [29,30,31]. The main advantages of our device with respect to the ones already presented in literature are the ability of performing simultaneous DCS and TD-NIRS measurements, and the lower acquisition time of 1 s. Indeed, He et al. [29] and Khalid et al. [31] presented hybrid devices that acquired sequentially TD-NIRS and DCS signals, with shutters placed in front of the two lasers and the TD-NIRS detector, to prevent cross-talk between the two modules. Moreover, the exposure time of DCS measurements was higher than 1 s: He et al. [29] showed DCS results obtained using 10 s of exposure time; Khalid et al. [31] collected data at temporal resolution of 300 ms, for a total acquisition time of 90 s; finally, Milejet al. [30] used a temporal resolution of 3 Hz (4.5 s moving average), and showed results integrating 30 s of measurements.

Simultaneous measurements of DCS and TD-NIRS modules were presented in 2019 by Giovannella et al. [12], with the BabyLux device: an hybrid system for hemodynamic monitoring of preterm neonates. Compared to the BabyLux, the hybrid device we presented in this work is characterized by a higher number of detection channels in the DCS module, which allows increasing the inter-fiber distance to 2.5 cm, with good signal to noise ratio; and by more innovative technologies in the TD-NIRS module [14].

Thanks to the double inter-fiber distances, the DCS device allows to discriminate shallow from deep tissue BFI (see Section 3.1). Due to safety reasons (according to EN-60825-1), the optical signal of the longer inter-fiber distance was divided in two injection points, so that the maximum power per unit area was always within the safety requirements.

In this work, we combined characterization measurements found in literature and added new ones to define our instrument performances. Like Giovannella et al. [12], we tested our device behavior over time, performing stability and repeatability measurements. The results reported in Section 3.1.1 and Section 3.1.2 show: high stability during long measurement (with negligible warm up time), and strong reproducibility of measurements over different days (with CV of about 2%). Results are in line with the state of the art DCS system [12]. The ability of our device in measuring $D_{b}$ variations was verified, by changing phantom temperature (as Carp et al. [10]) and viscosity (as Cortese et al. [24]). $D_{b}$ dependence from these two parameters was perfectly reproduced in accordance with theory [23] and previous findings [10,24].

Moreover, the effects of phantom optical properties, total detected count rate, and presence of TD-NIRS incoherent source on dynamic parameters β and $D_{b}$ were quantified. Optical parameters strongly influence $D_{b}$ estimation, and larger errors are related to reduced scattering coefficient variations (Figure 6), in accordance with [32]. Count rate increase (Figure 8) determined a reduction of $D_{b}$, which reached a plateau at 45 kcps, highlighting the importance of signal quality and equalization. Presence of incoherent TD-NIRS source causes reduction of β and increase of $D_{b}$ when the distance from DCS detection point is lower than 15 mm (Figure 10), remarking the importance of well-designed probe geometry.

Finally, the sensitivity of the DCS device to deep tissues was studied, highlighting the importance of the multi-distance device to discriminate influence of superficial layers. Measurements on bilayer phantom show a reduction of deep layer sensitivity when superficial layer thickness was increased (Figure 9); reaching an extremely low sensitivity (almost zero) when thickness of the superficial layer becomes roughly larger than a half of the inter-fiber distance.

In vivo measurements, reported in Section 3.1.9 and Section 3.1.10, highlight the ability of the device in following BF variations. Results reported in Figure 11 and Figure 12 are perfectly in accordance with literature [27,28], empowering the possibility of using our device for clinical investigations.

## 5. Conclusions

We presented a new multi-distance DCS device, integrated with a compact state-of-the-art TD-NIRS instrument. The hybrid device was systematically characterized, and the obtained results show good performances compared to other state-of-the-art DCS systems. Sensitivity to deep tissues was studied to highlight the importance of multiple inter-fiber distance to discriminate the effects of superficial tissues. From these results, also evident appears the need for a robust analysis model that considers the influence of superficial layers.

## Figures and Tables

**Table 1:** Optical properties µ_s_’ and µ_a_ measured on different liquid phantoms. The percentage error for optical properties and $D_{b}$ when, singularly, absorption and scattering were set to the average values among the phantoms.

Phantom #	µ’_s_ [cm^−1^]	µ_a_ [cm^−1^]	Error µ’_s_ [%]	Average Optical Values [cm^−1^]	Error µ_a_ [%]	**Error**$D_{b}$ [%]
0	10.91	0.047	0	0.11	−45	−23
1	10.83	0.089	0	0.11	−11	−4
2	10.84	0.130	0	0.11	23	7
3	10.85	0.159	0	0.11	45	12
4	6.84	0.094	−41	12.58	0	58
5	10.79	0.088	10	12.58	0	16
6	15.22	0.096	23	12.58	0	−41
7	17.72	0.097	41	12.58	0	−76

## Data Availability

Data underlying the results presented in this paper are not publicly available at this time but may be obtained from the corresponding authors upon reasonable request.
